# Animal models in neuroscience with alternative approaches: Evolutionary, biomedical, and ethical perspectives

**DOI:** 10.1002/ame2.12487

**Published:** 2024-10-07

**Authors:** Sabina Neziri, Ahmet Efe Köseoğlu, Gülsüm Deniz Köseoğlu, Buminhan Özgültekin, Nehir Özdemir Özgentürk

**Affiliations:** ^1^ Department of Molecular Biology and Genetics, Faculty of Art and Science Yıldız Technical University Istanbul Turkey; ^2^ COMED Therapeutics, Malta Life Sciences Park, San Gwann Malta; ^3^ Experimental Eye Research Institute Ruhr University Bochum Bochum Germany; ^4^ Department of Biomedical Engineering, Faculty of Engineering and Natural Sciences Acıbadem University Istanbul Turkey

**Keywords:** alternatives, animal models, biomedicine, ethics, evolution, neuroscience

## Abstract

Animal models have been a crucial tool in neuroscience research for decades, providing insights into the biomedical and evolutionary mechanisms of the nervous system, disease, and behavior. However, their use has raised concerns on several ethical, clinical, and scientific considerations. The welfare of animals and the 3R principles (replacement, reduction, refinement) are the focus of the ethical concerns, targeting the importance of reducing the stress and suffering of these models. Several laws and guidelines are applied and developed to protect animal rights during experimenting. Concurrently, in the clinic and biomedical fields, discussions on the relevance of animal model findings on human organisms have increased. Latest data suggest that in a considerable amount of time the animal model results are not translatable in humans, costing time and money. Alternative methods, such as in vitro (cell culture, microscopy, organoids, and micro physiological systems) techniques and in silico (computational) modeling, have emerged as potential replacements for animal models, providing more accurate data in a minimized cost. By adopting alternative methods and promoting ethical considerations in research practices, we can achieve the 3R goals while upholding our responsibility to both humans and other animals. Our goal is to present a thorough review of animal models used in neuroscience from the biomedical, evolutionary, and ethical perspectives. The novelty of this research lies in integrating diverse points of views to provide an understanding of the advantages and disadvantages of animal models in neuroscience and in discussing potential alternative methods.

## Graphical abstract

Animal models have played a key role in neuroscience research for years. Nevertheless, their usage brings up ethical and transability issues concerning humans. In the recent years, alternative approaches have been developed, containing a potential for replacement and reduction of animal experimenting, associated with more relatable results. This review covers the role the animal models have played so far, the ethical issues, and new replacing technologies.

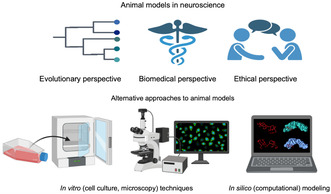

## 1 INTRODUCTION

**FIGURE 1:**
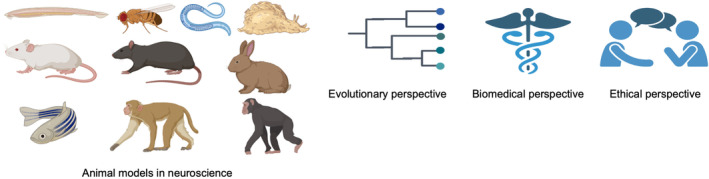
Animal models in neuroscience within evolutionary, biomedical, and ethical perspectives. The figure was created on BioRender (https://www.biorender.com).

Neuroscience is a field that studies the genetic, molecular, cellular, and physiological processes of the nervous system, including the brain, spinal cord, and peripheral neural system (PNS).^1^ Animal models have been a fundamental tool in neuroscience research for decades.^2^ The use of animal models in neuroscience dates to the ancient Egyptians, Greeks, and Romans.^3^ The first records of animal models in neuroscience included chickens, dogs, fish, pigeons, pigs, and mice, and then, frogs became widely used.^4^, ^5^ In the late 1800s, several invertebrate organisms started being used as model animals in research, such as nematodes, insects, planarians, mollusks, and crustaceans.^6^ In the 21st century, the most used animal models include rodents, nematodes, fruit flies, zebrafish, guinea pig, ferret, hamsters, chicken, nonhuman primates, and large domesticated animals.^2^, ^7^, ^8^ Animal models provide scientists with an approach to understand the mechanism and evolution of various neurological conditions, as well as to test different therapies.^9^ Some of the widely used animal models in neuroscience include primates (chimpanzees and macaques), rodents (mice and rats), zebrafish, salamanders, and *Aplysia*.^10^, ^11^, ^12^ These models have provided important insight into the nervous system, cognitive processes, neurodegenerative diseases, evolution, and drug development.^9^, ^10^, ^11^, ^12^ Even though no animal model can entirely recapitulate the human brain in a complete way because of the unique evolutionary history of all species,^13^ they provide a significant tool for making new discoveries and developing new therapies.^14^ Each model has its own advantages and disadvantages based on the research type.^15^ Moreover, the comparative method and One Health perspective connecting humans with other animals are increasing day by day to understand neurological basis and to solve common neurological diseases in terms of evolution and biomedicine^16^, ^17^ (Figure 1; Table 1).

**TABLE 1:** Animal models in neuroscience based on evolutionary and biomedical perspectives.

Animal model	Evolutionary perspective	Biomedical perspective	Reference
Chimpanzee (*Pan troglodytes*)	Human‐specific neurological changes	Nonhuman primate neurodegenerative disease model	18, 19, 20
Bonobo (*Pan paniscus*)	Human‐specific neurological changes	Nonhuman primate neurodegenerative disease model	18, 19, 21
Rhesus macaque (*Macaca mulatta*)	Mirror neurons, evolution of behavior and cognition	Neotenic brain development, PD, HD	22, 23, 24, 25, 26, 27
Rat (*Rattus norvegicus*)	Neuronal architecture, brain evolution, and evolution of neural tube closure patterns	Depression, anxiety disorders, and antidepressant drugs	28, 29, 30, 31, 32
Mouse (*Mus musculus*)	Neuronal architecture, brain evolution, and evolution of neural tube closure patterns	Humanized chimeric nervous system, HD, PD, Down syndrome, congenital hypomyelination treatment	32, 33, 34, 35, 36, 37, 38, 39, 40
European rabbit (*Oryctolagus cuniculus*)	Evolution of neural tube closure patterns	MS treatment, AD, neurological effects of vaccines and antiviral drugs in pregnant rabbits, anencephaly, and spina bifida	40, 41, 42, 43
Pig (*Sus scrofa*)	Evolution of neural tube closure patterns	HD, Neurofibromatosis type 1	40, 44, 45
Dog (*Canis lupus familiaris*)	Evolution of age‐related changes and brain atrophy	Multiple brain tumors (glioma and leptomeningeal sarcoma), epilepsy, spinal cord injury, stroke, and AD	17, 46, 47
Cat (*Felis catus*)	Evolution of age‐related changes and brain atrophy	Brain tumors, epilepsy, spinal cord injury, stroke, and AD	17, 46
Zebrafish (*Danio rerio*)	Vertebrate brain evolution, motor function, cognition	Epilepsy, schizophrenia, intellectual disability, autism spectrum disorders, depression, anxiety	11, 48, 49, 50, 51
Amphioxus (*Branchiostoma lanceolatum*)	Evolutionary origins of vertebrate brains, frontal brain‐like region	Neural tube and notochord regeneration	52, 53, 54, 55
Fruit fly (*Drosophila melanogaster*)	Conserved molecular neural pathways	AD, PD, neural tumors, CNS damage, and epilepsy	56, 57
Nematode (*Caenorhabditis elegans*)	Evolution of neural processes like circuit function, synaptic transmission, and minimal nervous system	Neurodegeneration, autism, and diseases such as PD and AD	58, 59, 60, 61, 62, 63, 64, 65
California sea slug (*Aplysia californica*)	Learning, short‐term and long‐term memory	AD	66, 67, 68

Abbreviations: AD, Alzheimer's disease; CNS, central nervous system; HD, Huntington's disease; MS, multiple sclerosis; PD, Parkinson's disease.

Despite their contributions and importance in research, the usage of animal models comes together with ethical concerns and specific limitations. Moral standards and laws related to animal testing are topics of ongoing discussion and discord.^69^ During experiments, model animals experience a poor quality of life, facing physical, mental, and physiological pain and distress.^70^ Ethically, the scientists are trying to reduce, refine, and replace the animal models (3Rs). Furthermore, the results obtained from animal experimenting are not always translatable in humans. In drug development, Food and Drug Administration (FDA) acknowledges that animal models don't predict the drug safety in humans.^71^ Almost 90% of the drug developments fail, one of the reasons being the failure of getting the same results in humans as in animal models.^72^, ^73^ In behavioral neuroscience, the results from animal experimenting fail to predict the outcomes in humans in more than 90% of the cases.^74^ In the case of neurodegenerative diseases, even though animal models have helped gain insights in several diseases, their usage in preclinical trials resulted in disappointment.^75^, ^76^ Moreover, the cost of animal research is high and delays the drug development process.^73^ These challenges have increased the interest in alternative approaches to animal models.

**FIGURE 2:**
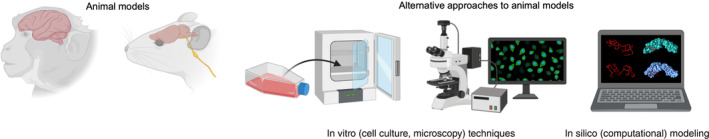
Alternative approaches to animal models in neuroscience. The figure was created on BioRender (https://www.biorender.com).

An increasing number of studies are focusing on alternative approaches contrary to animal model usage, including in vitro (cell culture and microscopy) techniques and in silico (computational) modeling in neuroscience studies^21^, ^77^, ^78^, ^79^, ^80^, ^81^, ^82^, ^83^, ^84^, ^85^, ^86^, ^87^, ^88^, ^89^, ^90^, ^91^, ^92^, ^93^, ^94^, ^95^, ^96^, ^97^, ^98^, ^99^ (Figure 2; Table 2). Advancements in technology have led to the development of innovative strategies such as organoids, microphysiological systems (organs on chips [OoCs]), induced pluripotent stem cells (iPSCs), advanced imaging methods, molecular docking, physiologically based pharmacokinetic (PBPK) models, and quantitative structure–activity relationship (QSAR) models.^100^, ^101^, ^102^, ^103^, ^104^

**TABLE 2:** Alternative approaches (in vitro and in silico) to animal models in neuroscience.

Approach	Disease model	Cell type	Reference
In vitro (3D cell culture and microscopy) technique In silico (computational) modeling	AD	Microglia, astrocyte Neural cells	81, 91
In vitro (cell culture and microscopy) technique	FTD	Peripheral blood mononuclear cells (PBMCs) Fibroblast cells	86, 87
In vitro (3D, organoid cell culture, and microscopy) technique In silico (computational) modeling	PD	SH‐SY5Y neuroblastoma cell line LUHMES	93, 96, 98
In vitro (cell culture and microscopy) technique In silico (computational) modeling	HD	Specific mutation‐carrying human embryonic stem cells	85, 99
In vitro (cell culture and functional neuroimaging) technique	Schizophrenia	Microglia	79, 95
In vitro (3D, organotypic ex vivo‐based cell culture and microscopy) technique	Peripheral nerve degeneration	Schwann cells Neuronal cells	^88^
In vitro (organotypic cell culture and microscopy) technique	MS	Human oligodendrocytes or oligodendrocyte precursor cells (OPCs) IPSC‐derived OPCs	21, 83, 89
In vitro (organ‐like 3D coculture system and microscopy) technique	ALS	IPSC‐derived cell lines Immortalized cell line	84, 90, 92
In silico (computational) modeling	Motor control and stroke	Brain and spinal cord neurons	^97^
In vitro (microfluidic cell culture and microscopy) technique	Axonal response to injury Synaptic formation and function Myelination Neuronal response to chemical inducements	CNS neurons and glial cells	77, 78, 80, 82

Abbreviations: AD, Alzheimer's disease; ALS, amyotrophic lateral sclerosis; CNS, central nervous system; FTD, frontotemporal dementia; HD, Huntington's disease; MS, multiple sclerosis; PD, Parkinson's disease.

The aim of this study is to summarize the use of animal models in neuroscience, highlighting their importance in evolutionary and biomedical research while also discussing the limitations and ethical concerns associated with these models (Figure 1; Table 1). Furthermore, alternative approaches, including in vitro techniques and in silico methods and the way these innovations are shaping neuroscience research, are also discussed (Figure 2; Table 2).

## 2 EVOLUTIONARY AND BIOMEDICAL PERSPECTIVES

Animal models have been used over the years in evolutionary studies.^105^ Vertebrates have evolved in more specialized tissues and complex neurological structure, including several nerve branches and a vascular system, which is highly branched, making them suitable models for human neurological conditions.^106^ Also, from a biomedical point of view, model animals have contributed greatly to the explanation and understanding of many mechanisms and development of new therapies (Figure 1; Table 1).

### 2.1 Mammals

The inability to directly study the origin of the complex brain and the central nervous system (CNS) is one of the biggest issues it faces. For instance, although there is much to be learned about evolutionary patterns and the genetic basis of mechanisms of different phenotypes in a variety of different animals, it is much more challenging and likely impossible to directly study the evolution of complex circuits in mammalian brains. Fortunately, two methods, the comparative method and the developmental method, can be used to determine the kinds of alterations that have taken place in the neocortex over the history of mammalian evolution and how those alterations were accomplished.^28^

The comparative approach examines similar features that all brains share, as well as derivations or specializations that have arisen in different mammalian brains because of adaptation to a unique lifestyle and environment, comparing distinct features of the neocortex of select mammals that ideally represent several phylogenetic branches of evolution, rather than just a few species such as monkeys, cats, and mice.^28^ The developmental approach studies the developmental mechanisms and their modifications that result in different adult phenotypes. The creation of the body and the brain is influenced by inherent genetic and activity‐dependent mechanisms, and changes to one or both cause species differences, according to studies of development.^28^

#### 2.1.1 Nonhuman primates

Because of their close phylogenetic relations with human and nonhuman primates, studies yield the most promising results that can be translated to humans. Similarities in the brain organization, social complexity, and involved cognitive abilities make this animal model very valuable in neuroscience research.^29^ The nonhuman primate model in brain research was apparent in 1981 Nobel Prize in Physiology or Medicine, when Wiesel and Hubel were awarded for their findings in the information processing by the visual system.^30^, ^107^ It was the macaque parkinsonian model that led to the discovery that brain stimulation of subthalamic brain structure was an effective therapy for the disease, improving the lives of 250 000 Parkinson's patients all over the world.^22^ Rhesus macaque (*Macaca mulatta*) was also used in neuroscience studies in terms of evolution of human brain, behavior, and cognition in addition to mirror neurons discovered for the first time in this animal model species.^23^, ^24^, ^25^ Among the nonhuman primate animal models, chimpanzees (*Pan troglodytes*) and bonobos (*Pan paniscus*) are the closest great ape relatives of humans, and they are the most important animal models to understand human‐specific neurological/genetic changes (cortex expansion, behavior, cognition, and complex language) and human neurodegenerative diseases (Alzheimer's disease [AD], Parkinson's disease [PD], Huntington's disease [HD], and dementia)^18^, ^19^ (Table 1).

Latest advances that come from monkey research include new therapies for stroke patients, improved understanding of the healthy brain cognitive functions and symptoms associated with psychiatric disorders, and the practice of brain–machine interfaces for reestablishing movement in paralyzed patients.^108^ Comparative genomic data resulted in human lineage‐specific (HLS) sequence identification.^31^ Because apes are humans' closest evolutionary relatives, the transgenic introduction of HLS in them contains a great potential to produce human phenotypes. For example, the transgenic rhesus monkeys that carry the *MCPH1* human gene variant showed human‐like neotenic brain development, leading to short reaction time and improved short‐term memory compared to the wild type.^26^ Another example is the transgenic rhesus macaque that developed HD, opening a way to better understanding of the disease and the development of new therapies.^27^ Such studies demonstrate the potential of transgenic nonhuman primates to provide important understandings in human neurodegenerative, cognitive, and social behavior disorders.

#### 2.1.2 Rodents

In recent years, 18%–20% of mammalian nervous system research has relied on rat (*Rattus norvegicus*) and mouse (*Mus musculus*) animal models, respectively, and rats and mice have been popular mammalian experimental models in various neuroscience research studies.^33^ They have been used to understand neuronal architecture and brain evolution in neuroscience studies.^34^ Nowadays, more than 200 000 mutant mouse strains can be generated among over 24 000 existing strains and over 209 000 altered mouse embryonic stem cells.^35^ Furthermore, rat strains are increasing, making rodents a remarkable genetic research model in biomedical research.^35^ Other advantages include their short life cycle, size, and maintenance simplicity. A difference has also been the generation of mouse–human chimaeras, which defines a tissue or organism that originated from at least two genetically diverse populations derived from different zygotes.^109^ An example is the creation of a humanized mouse chimeric nervous system that allows the study of human neural growth and the pathogenesis of several diseases.^32^ Chimeric mouse models have also been used to study HD, congenital hypomyelination treatment, AD, and Down syndrome^36^, ^37^, ^38^, ^39^ (Table 1).

Several aspects of PD, such as sleep disorders, behavior, and motor disability, can be modeled in rodents.^110^, ^111^ Depression, anxiety disorders, and antidepressant drugs have been studied in rats.^112^ Nonrodent animal models, such as *SOD1* mutations and aged nonhuman primates, have been studied for AD, but have not been effective, making rodents an important model.^76^ Nevertheless, preclinical research should be more rigorous, with acceptable replication and group sizes, an assessment of sex effects, and, if necessary, testing in several disease‐relevant models.^76^

#### 2.1.3 Other mammals

Rabbits (*Oryctolagus cuniculus*) can also be used in preclinical studies to identify fetal diseases in several parts of the brain after maternal exposure to different viruses and to identify active fetal viral infections after exposure during pregnancy.^41^ This model can be used for testing chemical substances for multiple sclerosis (MS) treatment, vaccines and antiviral drugs in pregnant rabbits and their probable effects on the neurological condition of embryos or fetuses, and the valuation of the effects of maternal anesthesia or surgery during pregnancy.^41^ In addition, rabbits are viewed as an ideal model for the development of maternal vaccines due to the embryological similarities with humans in the development of the CNS.^113^ Rabbits, having the amyloid amino acid sequence same as humans, present a nontransgenic model for AD study.^42^, ^43^ Furthermore, in human and rabbit embryos, the axial curvature increases throughout development, whereas the rate of neural tube closure reduces, and the parallels between these events could be used to examine illnesses like anencephaly and spina bifida that result from the failure of embryonic neural tube closure.^40^, ^114^ Moreover, from a comparative approach, evolution of neural tube closure patterns in rabbits was investigated in addition to various mammalian species, including rat, mouse, and pig^40^ (Table 1).

Pigs (*Sus scrofa*) are another mammalian animal models with both evolutionary (neural tube closure patterns) and biomedical (HD, neurofibromatosis type 1) perspectives in neuroscience studies with anatomical, physiological, and developmental similarities to humans.^40^, ^44^, ^45^ Cats (*Felis catus*) and dogs (*Canis lupus familiaris*) are remarkable companion animal models in neuroscience studies because of their bridge position between rodents and humans, and several human neurological diseases have also been reported in these companion animals, including brain tumors, epilepsy, spinal cord injury, stroke, and AD.^17^, ^46^ Moreover, there are evolutionary studies in neuroscience, including age‐related changes and brain atrophy in cats and dogs^17^ (Table 1).

### 2.2 Nonmammalian vertebrates

Zebrafish (*Danio rerio*) has become a widely used animal model in neuroscience because its transparent embryos and larvae allow for real‐time visualization, making it easy to breed and cost‐efficient. It is mostly used in brain function and disorder studies, behavior, drug‐induced conditions, and drug responses.^11^ Zebrafish are used as a model organism for several neurobehavioral disorders, such as depression,^48^ anxiety,^49^ and neurodevelopmental disorders, such as epilepsy, schizophrenia, intellectual disability, and autism spectrum disorders.^50^ Furthermore, behavior, motor functions during development, and cognition can be studied using zebrafish as an experimental model.^50^ Most importantly, zebrafish share with mammals the three basic divisions of a vertebrate brain: the forebrain, midbrain, and hindbrain.^51^ Zebrafish models in translational neuroscience have also gained a lot of importance because of their translational relevance to humans^115^ (Table 1).

### 2.3 Invertebrates

Comparisons among the vertebrate CNS and their invertebrate chordate relatives, such as tunicates and cephalochordates (amphioxus), give an understanding of the evolution of the vertebrate CNS. Nonetheless, there is still no consensus on the evolution of the chordate CNS.^116^, ^117^ The cephalochordate amphioxus remains to be a focus for the argumentative evolutionary origins of vertebrates after two centuries of discussion, with its phylogenetic position that makes amphioxus privileged to shed light on some of the key transitions of animal evolution, such as the origin of deuterostomes, the origin of chordates, or the origin of vertebrates.^52^ Gene expression patterns demonstrate that the frontal brain‐like region of the amphioxus nerve cord is locally subdivided in a similar way as the vertebrate brain.^53^, ^54^ Additionally, European amphioxus (*Branchiostoma lanceolatum*) has been used in regeneration studies, as its adults can regenerate structures like the neural tube and notochord.^55^ Comprehension of the evolution of the regenerative capacity in Metazoa depends on labeling the developmental and molecular foundations of organ regeneration in basal chordates, including amphioxus^55^ (Table 1).

Neuronal evolution research has depended on several animal models, and the first theory on neuron evolution was studied using *Hydra* as a model organism.^118^ Other studies and theories followed, using models such as medusas,^119^ sponge, and coelenterates (cnidarians and ctenophores).^120^ Gastropod mollusks, *Lymnae*, *Helix*, *Aplysia*, *Clione*, and *Tritonia*, include the most used animals during the 20th century.^121^ Molecular mechanisms and gene identification studies used mostly *Drosophila* as a model.^122^ *Caenorhabditis elegans*, Aplysia, and Drosophila have been used in discovering the molecular and cellular aspects of learning and memory.^123^ Significantly, fundamental memory attainment mechanisms of invertebrates seem to be organized in analogous principles as those of vertebrates.^124^

The fruit fly (*Drosophila melanogaster*) model is used for the study of molecular and cellular mechanisms related to different human disorders, mostly AD and PD, neural tumors, CNS damage, and epilepsy.^56^ Anatomically the difference between fruit flies and humans is evident, but the essential molecular neural pathways are still conserved.^57^ Nevertheless, conditions such as brain hemorrhage and brain infarcts cannot be studied in *Drosophila* because of the lack of vessels and the mostly primitive hemocytes made up of blood.^56^ However, using targeted gene expression, human proteins can be expressed in *Drosophila* animal models^125^ (Table 1).

The nematode (*C. elegans*) is used for neural processes like circuit function, synaptic transmission, autism, and diseases such as PD and AD.^58^, ^59^, ^60^, ^61^, ^62^, ^63^ *C. elegans* offers advantages such as short lifespan, sequenced genome, and a minimal nervous system, making it an ideal organism to study aging, deoxyribonucleic acid (DNA) repair, and neurodegeneration, as well as to study potential interventions to prevent or delay it.^64^, ^65^ It has a nervous system of around 300 neurons and a well‐defined synaptic connections network^126^, ^127^ (Table 1).

The California sea slug (*Aplysia californica*) is the popular experimental animal in neuroscience studied by Eric Kandel, who was awarded the Nobel Prize for Physiology or Medicine in 2000 for discovering the link between the brain and the environment in terms of learning short‐term and long‐term memory formations at the molecular level.^66^, ^67^ Aplysia offers a good system to study the molecular, physiological, and behavioral aspects of AD, with 898 potential protein orthologs to humans.^68^ Furthermore, *Aplysia* provides a neural model for the behavior and learning research in the neural circuit, molecular, cellular, and organism level.^128^, ^129^, ^130^ The aging effects in the sensory neurons' transcriptome can also be studied at a molecular level in this model animal^131^, ^132^ (Table 1).

## 3 ETHICAL PERSPECTIVE

Animals have been used for centuries in scientific research. The main aim of animal experiments was to improve animal and human health by understanding the mechanisms and sources of several diseases and disorders, developing and testing new therapies, treatments, surgical interventions, and medical appliances.^4^ Together with their big contribution to science, nevertheless, the use of animals in research raises concerns when it comes to the ethical implications in terms of animal welfare^133^.

Concerns about human and animal use in research were raised during the late 1800s and the start of the 20th century when medical research greatly expanded.^134^, ^135^ With the expose of numerous unethical research endeavors, such as the Tuskegee syphilis study and several medical tests performed on numerous prisoners during World War II, distrusts concerning the use of humans in research increased, and it was due to these violations, the Nuremberg Code, National Commission for the Protection of Human Subjects of Biomedical and Behavioral Research (1974), and the ensuing Belmont Report were founded.^136^ Today, these rules serve as a groundwork for the safety of human research subjects.

Ethical issues on animal experiments were initiated in 1959, with the 3Rs' principle of animal use.^16^ Government legislation, moral stands, and public opinions for a long time accompanied animal model usage. Government regulations constrain the researchers from causing pain, injury, or suffering during their animal model experiments.^137^ Each country has its own laws and guidelines for animal experimenting. In the United States of America, the Laboratory Animal Welfare Act was the first federal regulation related to animal research.^138^ The Guide for the Care and Use of Laboratory Animals must be followed by the scientists using animal models, and each research center must include an Institutional Animal Care and Use Committee (IACUC) and report its The Association for Assessment and Acreditation of Laboratory Animal Care (AAALAC) International accreditation.^138^ Public Health Service Policy on Humane Care and Use of Laboratory Animals (PHS Policy) is another guideline related to animal research.^138^ In the United Kingdom, the most significant legislation for animal experimenting is Animals (Scientific Procedures) Act 1986 (ASPA). The National Centre for the Replacement, Refinement and Reduction of Animals in Research (NC3Rs) is an organization that promotes the 3Rs.^138^, ^139^ The European Union legislation for animal welfare includes the Directive 2010/63/EU of the European Parliament and the Council.^140^ Other institutions and guidelines related to animal welfare and 3Rs over the world include Canadian Council on Animal Care (CCAC), the Australian Code for the Care and Use of Animals for Scientific Purposes, the Chinese National Guidelines (GB/T 35892‐20181) for the Ethical Review of Laboratory Animal Welfare, the Japanese Law for the Humane Treatment and Management of Animals.^141^, ^142^, ^143^, ^144^

Studies have confirmed that animals experience pain and distress^145^; thus even in gentle handling, they show hormonal stress markers and physiological changes.^146^ Furthermore, animals exhibit emotional states and pain responses like those of humans.^147^ Although the use of animal models played a significant role in numerous research studies, several data show that the results from animal experimentation did not relate or predict the human outcomes in clinical research.^148^, ^149^ This raised questions whether human diseases can be sufficiently mimicked in animals. Promising nonanimal methods are being developed, including the field of neurology.^150^ A significant turning point in the toxicology field occurred in 2007, when the U.S. National Research Council highlighted the necessity of in vitro and in silico (computational) methods to obtain more accurate data in the prediction of toxic effects in humans.^151^

Legislative, scientific, and ethical priorities are driving the replacement of animal testing by practices such as in vitro cell and tissue research, volunteer studies, physicochemical approaches, and computer modeling. Nonanimal methods are presently regarded as cutting‐edge ways that can address many of the problems of animal experiments. In vitro assays have avoided the use of a vast number of animals in testing drugs and chemicals. Research on neurological, reproductive, and dental issues has replaced certain animal models.^150^ To address significant areas of medical research and development, many researchers have started to exclusively use cell and tissue experiments, as well as human data. For example, to predict the immune response to a certain medicine or vaccination, an in vitro human immune system has been created in the field of vaccine testing and development.^152^, ^153^

There is constant discussion and debate about the moral principles and laws governing animal experimentation.^69^ Alternative methods that replace animal models can make it necessary to reexamine the rules and laws governing research, notwithstanding their great impact on science. The relevance of the research question and the viability of replacing animals with current methods from molecular and cell biology, genetics, biochemistry, and computational biology must be evaluated by researchers and oversight boards. Although none of these tools can reproduce an entire organism on their own, they do propose a mechanistic understanding of molecular activities. It is critical for researchers and reviewers to consider the anatomical, physiological, and genetic variations that may affect the applicability of findings, as well as changes in the clinical presentation and expression of diseases between species.^154^

## 4 ALTERNATIVE APPROACHES TO ANIMAL MODELS

Alternative approaches, such as in vitro and in silico techniques, have potential applications in neuroscience studies today and in the future, and they may eventually replace animal models^21^, ^77^, ^78^, ^79^, ^80^, ^81^, ^82^, ^83^, ^84^, ^85^, ^86^, ^87^, ^88^, ^89^, ^90^, ^91^, ^92^, ^93^, ^94^, ^95^, ^96^, ^97^, ^98^, ^99^ (Figure 2; Table 2). In vitro techniques include two‐dimensional (2D) and 3D cell culture models, which can be used for cell‐based assays and high‐throughput screening (HTS), organoids (3D structure with different cell types), microphysiological systems (organs on chips [OoCs]), and advanced imaging techniques.^71^ Up to date, the 2D cell culturing methods have been the most used because of their low cost and simplicity; however, they do not accurately reflect the fundamental physiology of real tissues.^155^ Controversially, 3D models can be adjusted to mimic the in vivo cell environment, leading to more accurate data on several diseases, drug discovery, stem cells research, metabolic profiling, and cell‐to‐cell interactions.^156^ Furthermore, the 3D models provide the possibility to study the organ dynamics using organoids, closing the gap among 2D cell culture techniques and animal models.^156^ For example, brain organoids recapitulate the tissue construction, the cell variety, and maturation, reducing the gap with in vivo features.^157^ OoCs is a new and innovative method in biomedical research. Brain‐on‐chip models can reproduce the essential functions of the organ in both the normal and pathophysiological settings.^158^ Other OoCs used in neuroscience include the neurovascular unit, blood–brain barrier, and nerve signal transduction chips.^159^

These methods have several advantages compared to animal models. In behavioral neuroscience, the results from animal experimenting fail to predict the outcomes in humans more than 90% of the cases.^74^ Animal models have weakened immune systems and lack the stroma–tumor interactions like humans.^160^ Finding concordance among animal models and clinical cancer trials is still difficult with the average percentage of concordant results being <8%.^161^, ^162^ Moreover, the cost of animal research is high and delays the drug development process.^73^ 3D models eliminate these problems making possible direct human testing.^156^ In the case of neurodegenerative diseases, even though animal models have helped gain insights in several diseases, their usage in preclinical trials resulted in failure.^75^, ^76^ Similarly, for HD, researchers used cell culture techniques in specific mutation‐carrying human embryonic stem cells and in silico (computational) modeling approaches.^85^, ^99^ In Schizophrenia, cell culture techniques targeting microglial cells and microscopy (functional neuroimaging) were applied to reveal the disease pathology.^79^, ^95^ Moreover, cell culture (3D and organotypic/organ‐like coculture) and microscopy techniques were also used for neurological diseases, such as peripheral nerve degeneration, MS, and amyotrophic lateral sclerosis (ALS).^21^, ^83^, ^84^, ^88^, ^89^, ^90^, ^92^ In vitro cell culture (microfluidic) and microscopy techniques were also applied for CNS neurons and glial cells to understand several situations, including axonal response to injury, synaptic formation and function, myelination, and neuronal response to chemical inducements.^77^, ^78^, ^80^, ^82^ Neural organoids have been generated from human pluripotent stem cells to mimic the developing brain, and when combined with machine learning they can be used to predict neural toxicity, promising potential use in drug and chemical safety testing.^163^ Furthermore, organoids have successfully used for creating models such as lissencephaly, Miller–Dieker syndrome,^164^ microcephaly, and cerebral cortex.^165^ Human induced pluripotent stem cells (hiPSC) and embryonic stem cells (ESC) have been used to construct the human blood–brain barrier.^166^ hiPSC has also been used to study genetic diseases such as AD^167^ and Rett syndrome.^168^

In silico (computational) methods include molecular docking, QSAR models, and physiologically based pharmacokinetic (PBPK) models.^102^, ^169^, ^170^ In silico methods have also been used in several evolutionary studies such as phylogenetic and structural analysis^171^, ^172^ and positive selection studies.^173^ In neuroscience, in silico methods have been used to study the evolution of neural networks linked to language.^174^ In a study, only in silico (computational) modeling approach was used to model brain and spinal cord neurons in motor control and stroke.^97^ The development of advanced statistical techniques and integrated experimentation design has reduced the use of animal models; for example, live embryos can now be replaced by in vitro embryonic stem cell testing.^175^

Nevertheless, the usage and applications of these alternative methods are associated with their own challenges. Organoid technology is associated with challenges such as expanding the cellular heterogeneity, simulating the micro‐ and matrix‐environment that cells encounter, creating reliable protocols that the in vitro organoid maturation remains fetal‐like.^176^ Furthermore, challenges associated to them include the absence of vascular system; for example as the organoid grows to a particular size, it becomes difficult for the cells in the center to get nutrition or excrete the metabolic waste.^177^ Other issues include the hydrogel biocompatibility and cost and creating the immune microenvironment of the human organism.^177^ Organoid use is associated with several ethical concerns, such as the stem cell source, cell donors' consent and privacy, moral and legal organoid status, the potential of attaining the human characteristics, gene editing usage, chimera creation, transplantation, patentability, commercialization, and storage.^178^ Specific guidelines and regulation may be needed to monitor the development in this field.^178^ In silico methods are associated with challenges such as an abundance of data, particularly important in the field of personalized medicine, and the need for developing biological systems' computational models (executable biology) to replicate the biological phenomena.^179^, ^180^ Furthermore, the deficiencies in standardized evaluation metrics, development of biomedical condition‐specific extraction methods, feature selection, interpretability, and the combination of several computational methods can impact performance.^181^

Alternative methods have many benefits, such as lower cost, less time requirements, and less complex experimental procedure.^182^ Adapting alternative methods to replace the animal models is associated with its own scientific and ethical challenges. Several researchers believe that development of alternative methods is still in its initial phase, and therefore they cannot utterly replace the animal models in preclinical research yet.^183^ At the moment, a limited number of these methods have been approved by the federal authorities, and extra funding will be required for the development and testing of these new methods.^183^

## 5 CONCLUSION

The advantages and disadvantages of animal models in neuroscience were discussed in terms of evolutionary and biomedical studies, and it was revealed that animal welfare and ethics should also be considered. In addition, in vitro (such as cell culture and microscopy) and in silico (computational) approaches have emerged as potential alternatives to animal models. By adopting alternative approaches and promoting ethical considerations in research practice, we can achieve these goals while maintaining our responsibility toward both humans and other animals. Furthermore, when designing an animal model study, we propose that evaluating in vitro and in silico analyses, and the using these alternative models, should be prerequisites for obtaining ethical approval in the future.

## 6 FUTURE DIRECTIONS

The intention is to increase the application of alternative methods in neuroscience research and improve animal welfare. The relevance of in vitro models for studying intricate cellular interactions and disease mechanisms can increase with further breakthroughs in this field, such as the creation of 3D cultures, organoids, and organ‐on‐a‐chip systems. Genetic engineering methods and patient‐derived cells could increase the usefulness of these models for medication screening and personalized therapy. Improved computational modeling methods, like machine learning algorithms and network‐based studies, will make it possible to forecast disease progression, treatment outcomes, and biomarker discovery with more accuracy. To leverage big data and convert computational discoveries into therapeutic applications, collaborations between neuroscientists, physicians, and computational biologists will be essential.

## AUTHOR CONTRIBUTIONS

**Sabina Neziri:** Conceptualization; formal analysis; investigation; methodology; writing – original draft. **Ahmet Efe Köseoğlu:** Conceptualization; formal analysis; investigation; methodology; supervision; writing – original draft; writing – review and editing. **Gülsüm Deniz Köseoğlu:** Formal analysis; investigation; writing – original draft. **Buminhan Özgültekin:** Formal analysis; investigation; writing – original draft. **Nehir Özdemir Özgentürk:** Supervision; writing – review and editing.

## FUNDING INFORMATION

This study was not funded.

## CONFLICT OF INTEREST STATEMENT

The authors declare no conflict of interest.

## ETHICS STATEMENT

No animal models were used in this study.

## References

[1] Neuroscience . Collection Development Guidelines of the National Library of Medicine. National Library of Medicine (U.S.); 2003. https://www.ncbi.nlm.nih.gov/books/NBK518776/

[2] Romanova EV , Sweedler JV . Animal model systems in neuroscience. ACS Chem Neurosci. 2018;9(8):1869‐1870. doi:10.1021/acschemneuro.8b00380 30107742

[3] Sandrone S . The amazing history of neuroscience. Frontiers for Young Minds; 2023. doi:10.3389/frym.2013.00014

[4] Ericsson AC , Crim MJ , Franklin CL . A brief history of animal modeling. Mo Med. 2013;110(3):201‐205. 23829102 PMC3979591

[5] Piccolino M , Bresadola M . Shocking Frogs: Galvani, Volta, and the Electric Origins of Neuroscience. Oxford University Press; 2013.

[6] Wilson‐Sanders SE . Invertebrate models for biomedical research, testing, and education. ILAR J. 2011;52(2):126‐152. doi:10.1093/ilar.52.2.126 21709307

[7] Hickman DL , Johnson J , Vemulapalli TH , Crisler JR , Shepherd R . Commonly used animal models. Principles of Animal Research for Graduate and Undergraduate Students; Elsevier; 2017:117‐175. doi:10.1016/B978-0-12-802151-4.00007-4

[8] Baxter VK , Griffin DE . Animal models. Viral Pathog (Third edition) 2016;125‐138. doi:10.1016/B978-0-12-800964-2.00010-0

[9] Chesselet M‐F , Carmichael ST . Animal models of neurological disorders. Neurotherapeutics. 2012;9(2):241‐244. doi:10.1007/s13311-012-0118-9 22460561 PMC3337025

[10] Arenas Gómez CM , Echeverri K . Salamanders: the molecular basis of tissue regeneration and its relevance to human disease. Curr Top Dev Biol. 2021;145:235‐275. doi:10.1016/bs.ctdb.2020.11.009 34074531 PMC8186737

[11] Kalueff AV , Stewart AM , Gerlai R . Zebrafish as an emerging model for studying complex brain disorders. Trends Pharmacol Sci. 2014;35(2):63‐75. doi:10.1016/j.tips.2013.12.002 24412421 PMC3913794

[12] Moroz LL . Aplysia. Curr Biol. 2011;21(2):R60‐R61. doi:10.1016/j.cub.2010.11.028 21256433 PMC4024469

[13] Setia H , Muotri AR . Brain organoids as a model system for human neurodevelopment and disease. Semin Cell Dev Biol. 2019;95:93‐97. doi:10.1016/j.semcdb.2019.03.002 30904636 PMC6755075

[14] Moon C . New insights into and emerging roles of animal models for neurological disorders. Int J Mol Sci. 2022;23(9):4957. doi:10.3390/ijms23094957 35563352 PMC9105220

[15] Jucker M . The benefits and limitations of animal models for translational research in neurodegenerative diseases. Nat Med. 2010;16(11):1210‐1214. doi:10.1038/nm.2224 21052075

[16] Mukherjee P , Roy S , Ghosh D , Nandi SK . Role of animal models in biomedical research: a review. Lab Anim Res. 2022;38:18. doi:10.1186/s42826-02200128-1 35778730 PMC9247923

[17] de Sousa AA , Rigby Dames BA , Graff EC , Mohamedelhassan R , Vassilopoulos T , Charvet CJ . Going beyond established model systems of Alzheimer's disease: companion animals provide novel insights into the neurobiology of aging. Commun Biol. 2023;6(1):655. 37344566 10.1038/s42003-023-05034-3 PMC10284893

[18] Lowenstine LJ , McManamon R , Terio KA . Comparative pathology of aging great apes: bonobos, chimpanzees, gorillas, and orangutans. Vet Pathol. 2016;53(2):250‐276. 26721908 10.1177/0300985815612154

[19] Pollen AA , Kilik U , Lowe CB , Camp JG . Human‐specific genetics: new tools to explore the molecular and cellular basis of human evolution. Nat Rev Genet. 2023;24:1‐25. 10.1038/s41576-022-00568-4 PMC9897628 36737647

[20] Grogan KE , Perry GH . Studying human and nonhuman primate evolutionary biology with powerful in vitro and in vivo functional genomics tools. Evol Anthropol Issues News Rev. 2020;29(3):143‐158. 10.1002/evan.21825 PMC10574139 32142200

[21] Bechler ME , Byrne L . CNS myelin sheath lengths are an intrinsic property of oligodendrocytes. Curr Biol. 2015;25(18):2411‐2416. 26320951 10.1016/j.cub.2015.07.056 PMC4580335

[22] DeLong MR , Benabid A‐L . Discovery of high‐frequency deep brain stimulation for treatment of Parkinson disease: 2014 Lasker award. JAMA. 2014;312(11):1093‐1094. doi:10.1001/jama.2014.11132 25198255

[23] Rizzolatti G , Fadiga L , Fogassi L , Gallese V . 14 from mirror neurons to imitation: facts and speculations. Imitative Mind Dev Evol Brain Bases. 2002;6:247‐266.

[24] Caggiano V , Fogassi L , Rizzolatti G , Thier P , Casile A . Mirror neurons differentially encode the peripersonal and extrapersonal space of monkeys. Science. 2009;324(5925):403‐406. 19372433 10.1126/science.1166818

[25] Calapai A , Berger M , Niessing M , et al. A cage‐based training, cognitive testing and enrichment system optimized for rhesus macaques in neuroscience research. Behav Res Methods. 2017;49:35‐45. 26896242 10.3758/s13428-016-0707-3 PMC5352800

[26] Shi L , Luo X , Jiang J , et al. Transgenic rhesus monkeys carrying the human MCPH1 gene copies show human‐like neoteny of brain development. Natl Sci Rev. 2019;6(3):480‐493. doi:10.1093/nsr/nwz043 34691896 PMC8291473

[27] Yang S‐H , Cheng P‐H , Banta H , et al. Towards a transgenic model of Huntington's disease in a non‐human primate. Nature. 2008;453(7197):7197‐7924. doi:10.1038/nature06975 PMC2652570 18488016

[28] Krubitzer L . In search of a unifying theory of complex brain evolution. Ann N Y Acad Sci. 2009;1156:44‐67. doi:10.1111/j.1749-6632.2009.04421.x 19338502 PMC2666944

[29] Capitanio JP , Emborg ME . Contributions of non‐human primates to neuroscience research. Lancet. 2008;371(9618):1126‐1135. doi:10.1016/S0140-6736(08)60489-4 18374844

[30] Barlow HB . David Hubel and Torsten Wiesel: their contributions towards understanding the primary visual cortex. Trends Neurosci. 1982;5:145‐152. doi:10.1016/0166-2236(82)90087-X

[31] Coors ME , Glover JJ , Juengst ET , Sikela JM . The ethics of using transgenic non‐human primates to study what makes us human. Nat Rev Genet. 2010;11(9):658‐662. doi:10.1038/nrg2864 20717156 PMC2995325

[32] Chen C , Kim W‐Y , Jiang P . Humanized neuronal chimeric mouse brain generated by neonatally engrafted human iPSC‐derived primitive neural progenitor cells. JCI Insight. 2016;1(19):e88632. doi:10.1172/jci.insight.88632 27882348 PMC5111502

[33] Żakowski W . Animal use in neurobiological research. Neuroscience. 2020;433:1‐10. doi:10.1016/j.neuroscience.2020.02.049 32156550

[34] Francis C , Natarajan S , Lee MT , et al. Divergence of RNA localization between rat and mouse neurons reveals the potential for rapid brain evolution. BMC Genomics. 2014;15(1):1‐18. 25301173 10.1186/1471-2164-15-883 PMC4203888

[35] Fahey JR , Katoh H , Malcolm R , Perez AV . The case for genetic monitoring of mice and rats used in biomedical research. Mamm Genome. 2013;24(3):89‐94. doi:10.1007/s00335-012-9444-9 23314661 PMC3627018

[36] Windrem MS , Schanz SJ , Guo M , et al. Neonatal chimerization with human glial progenitor cells can both remyelinate and rescue the otherwise lethally hypomyelinated shiverer mouse. Cell Stem Cell. 2008;2(6):553‐565. doi:10.1016/j.stem.2008.03.020 18522848 PMC3358921

[37] Osipovitch M , Asenjo Martinez A , Mariani JN , et al. Human ESC‐derived chimeric mouse models of Huntington's disease reveal cell‐intrinsic defects in glial progenitor cell differentiation. Cell Stem Cell. 2019;24(1):107‐122.e7. doi:10.1016/j.stem.2018.11.010 30554964 PMC6700734

[38] McQuade A , Kang YJ , Hasselmann J , et al. Gene expression and functional deficits underlie TREM2‐knockout microglia responses in human models of Alzheimer's disease. Nat Commun. 2020;11:5370. doi:10.1038/s41467-020-19227-5 33097708 PMC7584603

[39] Jin M , Xu R , Wang L , et al. Type‐iinterferon signaling drives microglial dysfunction and senescence in human iPSC models of down syndrome and Alzheimer's disease. Cell Stem Cell. 2022;29(7):1135‐1153.e8. doi:10.1016/j.stem.2022.06.007 35803230 PMC9345168

[40] Juriloff DM , Harris MJ . Insights into the etiology of mammalian neural tube closure defects from developmental, genetic and evolutionary studies. J Dev Biol. 2018;6(3):22. doi:10.3390/jdb6030022 30134561 PMC6162505

[41] de Almeida da Anunciação AR , Favaron PO , de Morais‐Pinto L , et al. Central nervous system development in rabbits (*Oryctolagus cuniculus* L. 1758). Anat Rec. 2021;304(6):1313‐1328. doi:10.1002/ar.24586 33480146

[42] Davidson JS , West RL , Kotikalapudi P , Maroun LE . Sequence and methylation in the beta/A4 region of the rabbit amyloid precursor protein gene. Biochem Biophys Res Commun. 1992;188(2):905‐911. doi:10.1016/0006-291x(92)91141-c 1445331

[43] Bitel CL , Kasinathan C , Kaswala RH , Klein WL , Frederikse PH . Amyloidβ and tau pathology of Alzheimer's disease induced by diabetes in a rabbit animal model. J Alzheimers Dis. 2012;32(2):291‐305. doi:10.3233/JAD-2012-120571 22785400

[44] White KA , Swier VJ , Cain JT , et al. A porcine model of neurofibromatosis type 1 that mimics the human disease. JCI Insight. 2018;3(12):e120402. 29925695 10.1172/jci.insight.120402 PMC6124439

[45] Yan S , Tu Z , Liu Z , et al. A huntingtin knockin pig model recapitulates features of selective neurodegeneration in Huntington's disease. Cell. 2018;173(4):989‐1002. 29606351 10.1016/j.cell.2018.03.005 PMC5935586

[46] Partridge B , Rossmeisl JH Jr . Companion animal models of neurological disease. J Neurosci Methods. 2020;331:108484. 31733285 10.1016/j.jneumeth.2019.108484 PMC6942211

[47] Rabotti GF , Grove AS , Sellers RL , Anderson WR . Induction of multiple brain tumours (gliomata and leptomeningeal sarcomata) in dogs by Rous sarcoma virus. Nature. 1966;209(5026):884‐886. 4288513 10.1038/209884a0

[48] Ziv L , Muto A , Schoonheim PJ , et al. An affective disorder in zebrafish with mutation of the glucocorticoid receptor. Mol Psychiatry. 2013;18(6):681‐691. doi:10.1038/mp.2012.64 22641177 PMC4065652

[49] Chakravarty S , Reddy BR , Sudhakar SR , et al. Chronic unpredictable stress (CUS)‐induced anxiety and related mood disorders in a zebrafish model: altered brain proteome profile implicates mitochondrial dysfunction. PLoS ONE. 2013;8(5):e63302. doi:10.1371/journal.pone.0063302 23691016 PMC3653931

[50] Sakai C , Ijaz S , Hoffman EJ . Zebrafish models of neurodevelopmental disorders: past, present, and future. Front Mol Neurosci. 2018;11:294. doi:10.3389/fnmol.2018.00294 30210288 PMC6123572

[51] Guo S . Using zebrafish to assess the impact of drugs on neural development and function. Expert Opin Drug Discov. 2009;4(7):715‐726. doi:10.1517/17460440902988464 19774094 PMC2747263

[52] Benito‐Gutiérrez È . A gene catalogue of the amphioxus nervous system. Int J Biol Sci. 2006;2(3):149‐160. 16763675 10.7150/ijbs.2.149 PMC1474150

[53] Holland LZ . Chordate roots of the vertebrate nervous system: expanding the molecular toolkit. Nat Rev Neurosci. 2009;10(10):736‐746. doi:10.1038/nrn2703 19738625

[54] Holland LZ , Carvalho JE , Escriva H , et al. Evolution of bilaterian central nervous systems: a single origin? EvoDevo. 2013;4(1):27. doi:10.1186/2041-9139-4-27 24098981 PMC3856589

[55] Somorjai IML , Somorjai RL , Garcia‐Fernàndez J , Escrivà H . Vertebrate‐like regeneration in the invertebrate chordate amphioxus. Proc Natl Acad Sci USA. 2012;109(2):517‐522. doi:10.1073/pnas.1100045109 22203957 PMC3258630

[56] Jeibmann A , Paulus W . Drosophila melanogaster as a model organism of brain diseases. Int J Mol Sci. 2009;10(2):407‐440. doi:10.3390/ijms10020407 19333415 PMC2660653

[57] Rubin GM , Lewis EB . A brief history of Drosophila's contributions to genome research. Science (New York, NY). 2000;287(5461):2216‐2218. doi:10.1126/science.287.5461.2216 10731135

[58] Miller KG , Alfonso A , Nguyen M , Crowell JA , Johnson CD , Rand JB . A genetic selection for *Caenorhabditis elegans* synaptic transmission mutants. Proc Natl Acad Sci USA. 1996;93(22):12593‐12598. doi:10.1073/pnas.93.22.12593 8901627 PMC38037

[59] Mahoney TR , Luo S , Nonet ML . Analysis of synaptic transmission in *Caenorhabditis elegans* using an aldicarb‐sensitivity assay. Nat Protoc. 2006;1(4):1772‐1777. doi:10.1038/nprot.2006.281 17487159

[60] Sengupta P , Samuel ADT . *C. elegans*: a model system for systems neuroscience. Curr Opin Neurobiol. 2009;19(6):637‐643. doi:10.1016/j.conb.2009.09.009 19896359 PMC2904967

[61] Calahorro F , Ruiz‐Rubio M . *Caenorhabditis elegans* as an experimental tool for the study of complex neurological diseases: Parkinson's disease, Alzheimer's disease and autism spectrum disorder. Invertebr Neurosci. 2011;11(2):73‐83. doi:10.1007/s10158-0110126-1 22068627

[62] Lindsay TH , Thiele TR , Lockery SR . Optogenetic analysis of synaptic transmission in the central nervous system of the nematode *Caenorhabditis elegans* . Nat Commun. 2011;2(1):1304. doi:10.1038/ncomms1304 PMC3935721 21556060

[63] Alexander AG , Marfil V , Li C . Use of *Caenorhabditis elegans* as a model to study Alzheimer's disease and other neurodegenerative diseases. Front Genet. 2014;5:279. doi:10.3389/fgene.2014.00279 25250042 PMC4155875

[64] Rapti G . A perspective on *C. elegans* neurodevelopment: from early visionaries to a booming neuroscience research. J Neurogenet. 2020;34(3–4):259‐272. doi:10.1080/01677063.2020.1837799 33446023

[65] Naranjo‐Galindo FJ , Ai R , Fang EF , Nilsen HL , SenGupta T . *C. elegans* as an animal model to study the intersection of DNA repair, aging and neurodegeneration. Front Aging. 2022;3:916118. doi:10.3389/fragi.2022.916118 35821838 PMC9261396

[66] Kandel ER . The molecular biology of memory storage: a dialogue between genes and synapses. Science. 2001;294(5544):1030‐1038. 11691980 10.1126/science.1067020

[67] Robertson M , Walter G . Eric Kandel and Aplysia californica: their role in the elucidation of mechanisms of memory and the study of psychotherapy. Acta Neuropsychiatr. 2010;22(4):195‐196. doi:10.1111/j.1601-5215.2010.00476.x

[68] Kron NS , Fieber LA . Aplysia neurons as a model of Alzheimer's disease: shared genes and differential expression. J Mol Neurosci. 2022;72(2):287‐302. doi:10.1007/s12031-021-01918-3 34664226 PMC8840921

[69] Kiani AK , Pheby D , Henehan G , et al. Ethical considerations regarding animal experimentation. J Prev Med Hyg. 2022;63(suppl. 3):E255‐E266. doi:10.15167/2421-4248/jpmh2022.63.2S3.2768 36479489 PMC9710398

[70] Morton DB . A model framework for the estimation of animal ‘suffering’: its use in predicting and retrospectively assessing the impact of experiments on animals. Animals. 2023;13(5):800. doi:10.3390/ani13050800 36899657 PMC10000069

[71] Vashishat A , Patel P , Das Gupta G , Das Kurmi B . Alternatives of animal models for biomedical research: a comprehensive review of modern approaches. Stem Cell Rev Rep. 2024;20(4):881‐899. doi:10.1007/s12015-024-10701-x 38429620

[72] Sun D , Gao W , Hu H , Zhou S . Why 90% of clinical drug development fails and how to improve it? Acta Pharm Sin B. 2022;12(7):3049‐3062. doi:10.1016/j.apsb.2022.02.002 35865092 PMC9293739

[73] Van Norman GA . Limitations of animal studies for predicting toxicity in clinical trials. JACC Basic Transl Sci. 2019;4(7):845‐854. doi:10.1016/j.jacbts.2019.10.008 31998852 PMC6978558

[74] Garner JP . The significance of meaning: why do over 90% of behavioral neuroscience results fail to translate to humans, and what can we do to fix it? ILAR J. 2014;55(3):438‐456. doi:10.1093/ilar/ilu047 25541546 PMC4342719

[75] Ransohoff RM . All (animal) models (of neurodegeneration) are wrong. Are they also useful? J Exp Med. 2018;215(12):2955‐2958. doi:10.1084/jem.20182042 30459159 PMC6279414

[76] Dawson TM , Golde TE , Lagier‐Tourenne C . Animal models of neurodegenerative diseases. Nat Neurosci. 2018;21(10):1370‐1379. doi:10.1038/s41593-018-0236-8 30250265 PMC6615039

[77] Taylor AM , Blurton‐Jones M , Rhee SW , Cribbs DH , Cotman CW , Jeon NL . A microfluidic culture platform for CNS axonal injury, regeneration and transport. Nat Methods. 2005;2(8):599‐605. doi:10.1038/nmeth777 16094385 PMC1558906

[78] Park J , Koito H , Li J , Han A . Microfluidic compartmentalized co‐culture platform for CNS axon myelination research. Biomed Microdevices. 2009;11(6):1145‐1153. doi:10.1007/s10544-009-9331-7 19554452 PMC2783938

[79] Gur RE , Gur RC . Functional magnetic resonance imaging in schizophrenia. Dialogues Clin Neurosci. 2010;12(3):333‐343. 20954429 10.31887/DCNS.2010.12.3/rgur PMC3181978

[80] Shi M , Majumdar D , Gao Y , et al. Glia co‐culture with neurons in microfluidic platforms promotes the formation and stabilization of synaptic contacts. Lab Chip. 2013;13(15):3008‐3021. doi:10.1039/c3lc50249j 23736663 PMC3712871

[81] Choi SH , Kim YH , Hebisch M , et al. A three‐dimensional human neural cell culture model of Alzheimer's disease. Nature. 2014;515(7526):274‐278. doi:10.1038/nature13800 25307057 PMC4366007

[82] Robertson G , Bushell TJ , Zagnoni M . Chemically induced synaptic activity between mixed primary hippocampal co‐cultures in a microfluidic system. Integr Biol. 2014;6(6):636‐644. doi:10.1039/c3ib40221e 24796407

[83] Di Ruscio A , Patti F , Welner RS , Tenen DG , Amabile G . Multiple sclerosis: getting personal with induced pluripotent stem cells. Cell Death Dis. 2015;6(7):e1806. 26158512 10.1038/cddis.2015.179 PMC4650727

[84] Smith I , Haag M , Ugbode C , et al. Neuronal‐glial populations form functional networks in a biocompatible 3D scaffold. Neurosci Lett. 2015;609:198‐202. 26493605 10.1016/j.neulet.2015.10.044

[85] Jacquet L , Neueder A , Földes G , et al. Three Huntington's disease specific mutation‐carrying human embryonic stem cell lines have stable number of CAG repeats upon in vitro differentiation into cardiomyocytes. PLoS ONE. 2015;10(5):e0126860. doi:10.1371/journal.pone.0126860 25993131 PMC4438866

[86] Rasmussen MA , Hjermind LE , Hasholt LF , et al. Induced pluripotent stem cells (iPSCs) derived from a patient with frontotemporal dementia caused by a P301L mutation in microtubule‐associated protein tau (MAPT). Stem Cell Res. 2016a;16(1):70‐74. 27345788 10.1016/j.scr.2015.12.008

[87] Rasmussen MA , Hjermind LE , Hasholt LF , et al. Induced pluripotent stem cells (iPSCs) derived from a patient with frontotemporal dementia caused by a R406W mutation in microtubule‐associated protein tau (MAPT). Stem Cell Res. 2016b;16(1):75‐78. 27345789 10.1016/j.scr.2015.12.006

[88] Geuna S , Raimondo S , Fregnan F , Haastert‐Talini K , Grothe C . In vitro models for peripheral nerve regeneration. Eur J Neurosci. 2016;43(3):287‐296. doi:10.1111/ejn.13054 26309051

[89] Madill M , Fitzgerald D , O'Connell KE , Dev KK , Shen S , FitzGerald U . In vitro and ex vivo models of multiple sclerosis. Drug Discov Today. 2016;21(9):1504‐1511. doi:10.1016/j.drudis.2016.05.018 27265771

[90] Krencik R , Seo K , van Asperen JV , et al. Systematic three‐dimensional coculture rapidly recapitulates interactions between human neurons and astrocytes. Stem Cell Rep. 2017;9(6):1745‐1753. 10.1016/j.stemcr.2017.10.026 PMC5785708 29198827

[91] Park J , Wetzel I , Marriott I , et al. A 3D human triculture system modeling neurodegeneration and neuroinflammation in Alzheimer's disease. Nat Neurosci. 2018;21(7):941‐951. 29950669 10.1038/s41593-018-0175-4 PMC6800152

[92] Pansarasa O , Bordoni M , Drufuca L , et al. Lymphoblastoid cell lines as a model to understand amyotrophic lateral sclerosis disease mechanisms. Dis Model Mech. 2018;11(3):dmm031625. 29419416 10.1242/dmm.031625 PMC5897724

[93] Lemos A , Melo R , Preto AJ , Almeida JG , Moreira IS , Dias Soeiro Cordeiro MN . In silico studies targeting G‐protein coupled receptors for drug research against Parkinson's disease. Curr Neuropharmacol. 2018;16(6):786‐848. doi:10.2174/1570159X16666180308161642 29521236 PMC6080095

[94] Sung JH , Wang Y , Shuler ML . Strategies for using mathematical modeling approaches to design and interpret multi‐organ microphysiological systems (MPS). APL Bioeng. 2019;3(2):021501. 31263796 10.1063/1.5097675 PMC6586554

[95] Sellgren CM , Gracias J , Watmuff B , et al. Increased synapse elimination by microglia in schizophrenia patient‐derived models of synaptic pruning. Nat Neurosci. 2019;22(3):374‐385. doi:10.1038/s41593-018-0334-7 30718903 PMC6410571

[96] Harischandra DS , Rokad D , Ghaisas S , et al. Enhanced differentiation of human dopaminergic neuronal cell model for preclinical translational research in Parkinson's disease. Biochim Biophys Acta Mol basis Dis. 2020;1866(4):165533. doi:10.1016/j.bbadis.2019.165533 31442530 PMC7010568

[97] Allegra Mascaro AL , Falotico E , Petkoski S , et al. Experimental and computational study on motor control and recovery after stroke: toward a constructive loop between experimental and virtual embodied neuroscience. Front Syst Neurosci. 2020;14:31. 32733210 10.3389/fnsys.2020.00031 PMC7359878

[98] Alrashidi H , Eaton S , Heales S . Biochemical characterization of proliferative and differentiated SH‐SY5Y cell line as a model for Parkinson's disease. Neurochem Int. 2021;145:105009. doi:10.1016/j.neuint.2021.105009 33684546

[99] Kohli H , Kumar P , Ambasta RK . In silico designing of putative peptides for targeting pathological protein Htt in Huntington's disease. Heliyon. 2021;7(2):e06088. doi:10.1016/j.heliyon.2021.e06088 33659724 PMC7890153

[100] Kim EA , Jung KC , Sohn UD , Im C . Quantitative structure activity relationship between diazabicyclo [4.2.0] octanes derivatives and nicotinic acetylcholine receptor agonists. Korean J Physiol Pharmacol. 2009;13(1):55‐59. doi:10.4196/kjpp.2009.13.1.55 19885027 PMC2766720

[101] Choquet D , Sainlos M , Sibarita JB . Advanced imaging and labelling methods to decipher brain cell organization and function. Nat Rev Neurosci. 2021;22(4):237‐255. 33712727 10.1038/s41583-021-00441-z

[102] Sánchez‐Dengra B , Gonzalez‐Alvarez I , Bermejo M , Gonzalez‐Alvarez M . Physiologically based pharmacokinetic (PBPK) modeling for predicting brain levels of drug in rat. Pharmaceutics. 2021;13(9):1402. doi:10.3390/pharmaceutics13091402 34575476 PMC8471455

[103] Sunildutt N , Parihar P , Chethikkattuveli Salih AR , Lee SH , Choi KH . Revolutionizing drug development: harnessing the potential of organ‐on‐chip technology for disease modeling and drug discovery. Front Pharmacol. 2023;14:1139229. doi:10.3389/fphar.2023.1139229 37180709 PMC10166826

[104] Köseoğlu AE , Zerin A , Tunç İ , et al. Comparing the impact of wild type and derived DBP allelic variants detected in the Turkish population on serum vitamin D levels by bioinformatics analysis. Hum Nutr Metab. 2024;36:200263.

[105] Kellogg EA , Shaffer HB . Model organisms in evolutionary studies. Syst Biol. 1993;42(4):409‐414. doi:10.2307/2992481

[106] Zacchigna S , de Almodovar CR , Carmeliet P . Similarities between angiogenesis and neural development: what small animal models can tell us. Curr Top Dev Biol. 2007;80:1‐55. doi:10.1016/S0070-2153(07)80001-9 17950371

[107] Hubel DH , Wiesel TN . Receptive fields, binocular interaction and functional architecture in the cat's visual cortex. J Physiol. 1962;160(1):106‐154. 14449617 10.1113/jphysiol.1962.sp006837 PMC1359523

[108] Mitchell AS , Thiele A , Petkov CI , et al. Continued need for non‐human primate neuroscience research. Curr Biol. 2018;28(20):R1186‐R1187. doi:10.1016/j.cub.2018.09.029 30352184 PMC6246749

[109] Morata Tarifa C , López Navas L , Azkona G , Sánchez Pernaute R . Chimeras for the twenty‐first century. Crit Rev Biotechnol. 2020;40(3):283‐291. doi:10.1080/07388551.2019.1679084 32054356

[110] Meredith GE , Kang UJ . Behavioral models of Parkinson's disease in rodents: a new look at an old problem. Mov Disord. 2006;21(10):1595‐1606. doi:10.1002/mds.21010 16830310

[111] Medeiros DDC , Lopes Aguiar C , Moraes MFD , Fisone G . Sleep disorders in rodent models of Parkinson's disease. Front Pharmacol. 2019;10:1414. doi:10.3389/fphar.2019.01414 31827439 PMC6892229

[112] Bondi CO , Rodriguez G , Gould GG , Frazer A , Morilak DA . Chronic unpredictable stress induces a cognitive deficit and anxiety‐like behavior in rats that is prevented by chronic antidepressant drug treatment. Neuropsychopharmacology. 2008;33(2):320‐331. doi:10.1038/sj.npp.1301410 17406647

[113] Peng X , Knouse JA , Hernon KM . Rabbit models for studying human infectious diseases. Comp Med. 2015;65(6):499‐507. 26678367 PMC4681244

[114] Peeters MCE , Hekking JWM , van Straaten HWM , Shum ASW , Copp AJ . Relationship between altered axial curvature and neural tube closure in normal and mutant (curly tail) mouse embryos. Anat Embryol. 1996;193(2):123‐130. doi:10.1007/BF00214703 8742053

[115] Stewart AM , Braubach O , Spitsbergen J , Gerlai R , Kalueff AV . Zebrafish models for translational neuroscience research: from tank to bedside. Trends Neurosci. 2014;37(5):264‐278. doi:10.1016/j.tins.2014.02.011 24726051 PMC4039217

[116] Holland LZ . Chapter 4—Invertebrate origins of vertebrate nervous systems. In: Kaas JH , ed. Evolutionary Neuroscience. 2nd ed. Academic Press; 2020:51‐73. doi:10.1016/B978-0-12-820584-6.00004-0

[117] Lacalli T . An evolutionary perspective on chordate brain organization and function: insights from amphioxus, and the problem of sentience. Philos Trans R Soc Lond Ser B Biol Sci. 2021;377(1844):20200520. doi:10.1098/rstb.2020.0520 34957845 PMC8710876

[118] Kleinenberg N . Hydra: Eine Anatomisch‐entwicklungsgeschichtliche Untersuchung. W. Engelmann; 1872.

[119] Hertwig O , Hertwig R . Das Nervensystem und die Sinnesorgane der medusen. Vogel; 1878.

[120] Parker GH . The Elementary Nervous System; 1919. https://philpapers.org/rec/PARTEN‐2

[121] Moroz LL . On the independent origins of complex brains and neurons. Brain Behav Evol. 2009;74(3):177‐190. doi:10.1159/000258665 20029182 PMC2855278

[122] Hirth F , Kammermeier L , Frei E , Walldorf U , Noll M , Reichert H . An urbilaterian origin of the tripartite brain: developmental genetic insights from drosophila. Development. 2003;130(11):2365‐2373. doi:10.1242/dev.00438 12702651

[123] Rahmani A , Chew YL . Investigating the molecular mechanisms of learning and memory using *Caenorhabditis elegans* . J Neurochem. 2021;159(3):417‐451. doi:10.1111/jnc.15510 34528252

[124] Van Damme S , De Fruyt N , Watteyne J , et al. Neuromodulatory pathways in learning and memory: lessons from invertebrates. J Neuroendocrinol. 2021;33(1):e12911. doi:10.1111/jne.12911 33350018

[125] Brand AH , Perrimon N . Targeted gene expression as a means of altering cell fates and generating dominant phenotypes. Development. 1993;118(2):401‐415. doi:10.1242/dev.118.2.401 8223268

[126] White JG , Southgate E , Thomson JN , Brenner S . The structure of the nervous system of the nematode *Caenorhabditis elegans* . Philos Trans R Soc Lond Ser B Biol Sci. 1986;314(1165):1‐340. doi:10.1098/rstb.1986.0056 22462104

[127] Cook SJ , Jarrell TA , Brittin CA , et al. Whole‐animal connectomes of both *Caenorhabditis elegans* sexes. Nature. 2019;571(7763):63‐71. doi:10.1038/s41586-019-1352-7 31270481 PMC6889226

[128] Bailey CH , Castellucci VF , Koester J , Chen M . Behavioral changes in aging Aplysia: a model system for studying the cellular basis of age‐impaired learning, memory, and arousal. Behav Neural Biol. 1983;38(1):70‐81. doi:10.1016/S0163-1047(83)90399-0 6626101

[129] Baxter DA , Byrne JH . Feeding behavior of Aplysia: a model system for comparing cellular mechanisms of classical and operant conditioning. Learn Mem. 2006;13(6):669‐680. doi:10.1101/lm.339206 17142299

[130] Moroz LL , Edwards JR , Puthanveettil SV , et al. Neuronal transcriptome of Aplysia: neuronal compartments and circuitry. Cell. 2006;127(7):1453‐1467. doi:10.1016/j.cell.2006.09.052 17190607 PMC4024467

[131] Moroz LL , Kohn AB . Single‐neuron transcriptome and methylome sequencing for epigenomic analysis of aging. In: Tollefsbol TO , ed. Biological Aging: Methods and Protocols. Humana Press; 2013:323‐352. doi:10.1007/978-1-62703-556-9_21 PMC4045448 23929113

[132] Greer JB , Schmale MC , Fieber LA . Whole‐transcriptome changes in gene expression accompany aging of sensory neurons in Aplysia californica. BMC Genomics. 2018;19(1):529. doi:10.1186/s12864-018-4909-1 29996779 PMC6042401

[133] Levy N . The use of animal as models: ethical considerations. Int J Stroke. 2012;7(5):440‐442. doi:10.1111/j.1747-4949.2012.00772.x 22712743

[134] Gossel PP . William Henry Welch and the antivivisection legislation in the District of Columbia, 1896–1900. J Hist Med Allied Sci. 1985;40(4):397‐419. doi:10.1093/jhmas/40.4.397 3905947

[135] Lederer S , Davis AB . Subjected to science: human experimentation in America before the second world war. Hist Rev New Books. 1995;24(1):13. doi:10.1080/03612759.1995.9949143 11618486

[136] Ferdowsian HR , Beck N . Ethical and scientific considerations regarding animal testing and research. PLoS ONE. 2011;6(9):e24059. doi:10.1371/journal.pone.0024059 21915280 PMC3168484

[137] VandeWoude S , Rollin BE . Practical Considerations in Regenerative Medicine Research: IACUCs, Ethics, and the Use of Animals in Stem Cell Studies; 2010. https://www.wellbeingintlstudiesrepository.org/bioamres/4 10.1093/ilar.51.1.82 20075500

[138] National Research Council (US) Committee to Update Science, Medicine, and Animals . Regulation of Animal Research. National Academies Press (US); 2004. https://www.ncbi.nlm.nih.gov/books/NBK24650/ 20669472

[139] Singh J . The national centre for the replacement, refinement, and reduction of animals in research. J Pharmacol Pharmacother. 2012;3(1):87‐89. 22368436 PMC3284057

[140] Marinou KA , Dontas IA . European Union legislation for the welfare of animals used for scientific purposes: areas identified for further discussion. Animals. 2023;13(14):2367. doi:10.3390/ani13142367 37508144 PMC10376073

[141] Griffin G . Establishing a three Rs Programme at the Canadian Council on animal care. Altern Lab Anim. 2009;37(suppl. 2):63‐67. doi:10.1177/026119290903702S09 20105015

[142] Hubrecht R . Revised Australian code for the care and use of animals for scientific purposes. Anim Welf. 2013;22(4):491. doi:10.1017/S0962728600005674

[143] MacArthur Clark JA , Sun D . Guidelines for the ethical review of laboratory animal welfare People's Republic of China National Standard GB/T 35892‐2018 [issued 6 February 2018 effective from 1 September 2018]. Anim Models Expe Med. 2020;3(1):103‐113. doi:10.1002/ame2.12111 PMC7167230 32318667

[144] National Research Council; Division on Earth and Life Studies; Institute for Laboratory Animal Research; International Workshop on the Development of Science‐Based Guidelines for Laboratory Animal Care Program Committee . The Development of Science‐Based Guidelines for Laboratory Animal Care: Proceedings of the November 2003 International Workshop. National Academies Press; 2004. 20669462

[145] Gregory NG . Physiology and Behaviour of Animal Suffering; n.d. Accessed April 9, 2023. https://books.google.com/books/about/Physiology_and_Behaviour_of_Animal_Suffe.html?id=0bOZocGJMaAC

[146] Balcombe JP , Barnard ND , Sandusky C . Laboratory Routines Cause Animal Stress. American Association for Laboratory Animal Science; 2004. https://www.ingentaconnect.com/content/aalas/jaalas/2004/00000043/00000006/art00009 15669134

[147] McMillan FD . Mental Health and Well‐being in Animals, 2nd ed. n.d. Accessed April 9, 2023. https://books.google.com/books/about/Mental_Health_and_Well_being_in_Animals.html?id=Lge9DwAAQBAJ

[148] Hackam DG , Redelmeier DA . Translation of research evidence from animals to humans. JAMA. 2006;296(14):1727‐1732. doi:10.1001/jama.296.14.1731 17032985

[149] Perel P , Roberts I , Sena E , et al. Comparison of treatment effects between animal experiments and clinical trials: systematic review. Br Med J. 2007;334(7586):197. doi:10.1136/bmj.39048.407928.BE 17175568 PMC1781970

[150] Langley G , Evans T , Holgate ST , Jones A . Replacing animal experiments: choices, chances and challenges. BioEssays. 2007;29(9):918‐926. doi:10.1002/bies.20628 17688239

[151] National Research Council, Division on Earth and Life Studies, Institute for Laboratory Animal Research, Board on Environmental Studies and Toxicology, Committee on Toxicity Testing and Assessment of Environmental Agents . Toxicity Testing in the 21st Century; n.d. Accessed April 18, 2023. https://books.google.com/books/about/Toxicity_Testing_in_the_21st_Century.html?id=3AWfAwAAQBAJ

[152] Byers AM , Tapia TM , Sassano ER , Wittman V . In vitro antibody response to tetanus in the MIMIC system is a representative measure of vaccine immunogenicity. Biologicals. 2009;37(3):148‐151. doi:10.1016/j.biologicals.2009.02.018 19272794

[153] Higbee RG , Byers AM , Dhir V , et al. An immunologic model for rapid vaccine assessment—a clinical trial in a test tube. Altern Lab Anim. 2009;37(suppl. 1):19‐27. doi:10.1177/026119290903701S05 19807200

[154] Akhtar A . The flaws and human harms of animal experimentation. Camb Q Healthc Ethics. 2015;24(4):407‐419. doi:10.1017/S0963180115000079 26364776 PMC4594046

[155] Hoarau‐Véchot J , Rafii A , Touboul C , Pasquier J . Halfway between 2D and animal models: are 3D cultures the ideal tool to study cancer‐microenvironment interactions? Int J Mol Sci. 2018;19(1):181. doi:10.3390/ijms19010181 29346265 PMC5796130

[156] Jensen C , Teng Y . Is it time to start transitioning from 2D to 3D cell culture? Front Mol Biosci. 2020;7:33. doi:10.3389/fmolb.2020.00033 32211418 PMC7067892

[157] Chiaradia I , Lancaster MA . Brain organoids for the study of human neurobiology at the interface of in vitro and in vivo. Nat Neurosci. 2020;23(12):1496‐1508. doi:10.1038/s41593-020-00730-3 33139941

[158] Amirifar L , Shamloo A , Nasiri R , et al. Brain‐on‐a‐chip: recent advances in design and techniques for microfluidic models of the brain in health and disease. Biomaterials. 2022;285:121531. doi:10.1016/j.biomaterials.2022.121531 35533441

[159] Li Z , Zhao Y , Lv X , Deng Y . Integrated brain on a chip and automated organ‐on‐chips systems. Interdiscip Med. 2023;1(1):e20220002. doi:10.1002/INMD.20220002

[160] Cekanova M , Rathore K . Animal models and therapeutic molecular targets of cancer: utility and limitations. Drug Des Devel Ther. 2014;8:1911‐1922. doi:10.2147/DDDT.S49584 PMC4206199 25342884

[161] Voskoglou‐Nomikos T , Pater JL , Seymour L . Clinical predictive value of the in vitro cell line, human xenograft, and mouse allograft preclinical cancer models. Clin Cancer Res. 2003;9(11):4227‐4239. 14519650

[162] Mak IW , Evaniew N , Ghert M . Lost in translation: animal models and clinical trials in cancer treatment. Am J Transl Res. 2014;6(2):114‐118. 24489990 PMC3902221

[163] Schwartz MP , Hou Z , Propson NE , et al. Human pluripotent stem cell‐derived neural constructs for predicting neural toxicity. Proc Natl Acad Sci USA. 2015;112:12516‐12521. doi:10.1073/pnas.1516645112 26392547 PMC4603492

[164] Bershteyn M , Nowakowski TJ , Pollen AA , et al. Human iPSC‐derived cerebral organoids model cellular features of lissencephaly and reveal prolonged mitosis of outer radial glia. Cell Stem Cell. 2017;20(4):435‐449.e4. doi:10.1016/j.stem.2016.12.007 28111201 PMC5667944

[165] Lancaster MA , Renner M , Martin C‐A , et al. Cerebral organoids model human brain development and microcephaly. Nature. 2013;501(7467):373‐379. doi:10.1038/nature12517 23995685 PMC3817409

[166] Lippmann ES , Al‐Ahmad A , Palecek SP , Shusta EV . Modeling the blood–brain barrier using stem cell sources. Fluids Barriers CNS. 2013;10(1):2. doi:10.1186/2045-8118-10-2 23305164 PMC3564868

[167] Israel MA , Yuan SH , Bardy C , et al. Probing sporadic and familial Alzheimer's disease using induced pluripotent stem cells. Nature. 2012;482(7384):216‐220. doi:10.1038/nature10821 22278060 PMC3338985

[168] Marchetto MCN , Carromeu C , Acab A , et al. A model for neural development and treatment of Rett syndrome using human induced pluripotent stem cells. Cell. 2010;143(4):527‐539. doi:10.1016/j.cell.2010.10.016 21074045 PMC3003590

[169] El Aissouq A , Bouachrine M , Ouammou A , Khalil F . Homology modeling, virtual screening, molecular docking, molecular dynamic (MD) simulation, and ADMET approaches for identification of natural anti‐Parkinson agents targeting MAO‐B protein. Neurosci Lett. 2022;786:136803. doi:10.1016/j.neulet.2022.136803 35842207

[170] Patel HM , Noolvi MN , Sharma P , et al. Quantitative structure–activity relationship (QSAR) studies as strategic approach in drug discovery. Med Chem Res. 2014;23(12):4991‐5007. doi:10.1007/s00044-014-1072-3

[171] Cherry AL , Wheeler MJ , Mathisova K , Di Miceli M . In silico analyses of the involvement of GPR55, CB1R and TRPV1: response to THC, contribution to temporal lobe epilepsy, structural modeling and updated evolution. Front Neuroinform. 2024;18:1294939. doi:10.3389/fninf.2024.1294939 38404644 PMC10894036

[172] Köseoğlu AE , Paltacı S , Can H , et al. Applicability evaluation of mtDNA based molecular identification in mosquito species/subspecies/biotypes collected from Thessaloniki, Greece. Vet Parasitol Reg Stud Rep. 2023;41:100869. doi:10.1016/j.vprsr.2023.100869 37208079

[173] Álvarez‐Carretero S , Kapli P , Yang Z . Beginner's guide on the use of PAML to detect positive selection. Mol Biol Evol. 2023;40(4):msad041. doi:10.1093/molbev/msad041 37096789 PMC10127084

[174] Szathmáry E , Szathmáry Z , Ittzés P , et al. In silico evolutionary developmental neurobiology and the origin of natural language. In: Lyon C , Nehaniv CL , Cangelosi A , eds. Emergence of Communication and Language. Springer; 2007:151‐187. doi:10.1007/978-1-84628-779-4_8

[175] Husain A , Meenakshi DU , Ahmad A , Shrivastava N , Khan SA . A review on alternative methods to experimental animals in biological testing: recent advancement and current strategies. J Pharm Bioallied Sci. 2023;15(4):165‐171. doi:10.4103/jpbs.jpbs_380_23 38235048 PMC10790740

[176] Lou Y‐R , Leung AW . Next generation organoids for biomedical research and applications. Biotechnol Adv. 2018;36(1):132‐149. doi:10.1016/j.biotechadv.2017.10.005 29056474

[177] Huang Y , Huang Z , Tang Z , et al. Research progress, challenges, and breakthroughs of organoids as disease models. Front Cell Dev Biol. 2021;9:740574. doi:10.3389/fcell.2021.740574 34869324 PMC8635113

[178] Mollaki V . Ethical challenges in organoid use. Biotech. 2021;10(3):12. doi:10.3390/biotech10030012 35822766 PMC9245480

[179] Fisher J , Henzinger TA . Executable cell biology. Nat Biotechnol. 2007;25(11):1239‐1249. doi:10.1038/nbt1356 17989686

[180] Jean‐Quartier C , Jeanquartier F , Jurisica I , Holzinger A . In silico cancer research towards 3R. BMC Cancer. 2018;18(1):408. doi:10.1186/s12885-018-4302-0 29649981 PMC5897933

[181] Houssein EH , Hosney ME , Emam MM , Younis EMG , Ali AA , Mohamed WM . Soft computing techniques for biomedical data analysis: open issues and challenges. Artif Intell Rev. 2023;56(2):2599‐2649. doi:10.1007/s10462-023-10585-2

[182] Park G , Rim YA , Sohn Y , Nam Y , Ju JH . Replacing animal testing with stem cell‐organoids: advantages and limitations. Stem Cell Rev Rep. 2024;20(6):1375‐1386. doi:10.1007/s12015-024-10723-5 38639829 PMC11319430

[183] Mahalmani V , Prakash A , Medhi B . Do alternatives to animal experimentation replace preclinical research? Indian J Pharm. 2023;55(2):71‐75. doi:10.4103/ijp.ijp_223_23 PMC10335642 37313932

